# Secondary Metabolite Plasticity in *Eclipta prostrata* (L.) L. (Asteraceae) under Environmental and Biological Stressors

**DOI:** 10.1021/acsomega.5c08697

**Published:** 2025-12-15

**Authors:** Sara T. D. da Fonseca, Ana M. S. Pereira, Norberto P. Lopes

**Affiliations:** † Núcleo de Pesquisa em Produtos Naturais e Sintéticos (NPPNS), Department of Biomolecular Sciences, Faculty of Pharmaceutical Sciences of Ribeirão Preto, 28133 University of São Paulo, Ribeirão Preto 14040-903, São Paulo, Brazil; ‡ Department of Plant Biotechnology, 42496 University of Ribeirão Preto (UNAERP), Ribeirão Preto 14096-900, São Paulo, Brazil

## Abstract

This study examined how light exposure, soil composition, and plant associations influence biomass and the accumulation of secondary metabolites in *Eclipta prostrata* (L.) L. (Asteraceae), a medicinal plant with significant ethnopharmacological value. Plants were grown under controlled conditions across different soil types and shade and in association with *Arachis repens* Hando or intraspecific association. Metabolite profiles were analyzed using HPLC-DAD and HPLC-DAD-ESI-MS/MS. The results revealed that light exposure, soil quality (especially Carolina soil), and plant associations significantly enhanced the production of key secondary metabolites. Interestingly, metabolite accumulation was not directly correlated with biomass, highlighting the influence of abiotic and biotic factors on the plant’s phytochemical plasticity.

## Graphical abstract

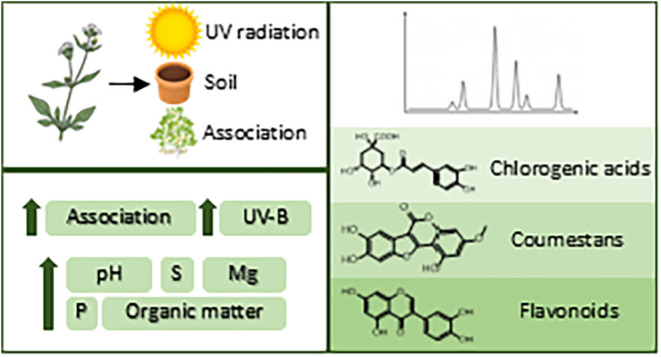

## Introduction

*Eclipta prostrata* (L.) L. (Asteraceae) (Figure 1) is a medicinal plant widely distributed across tropical and subtropical regions of Asia, Africa, and South America, with occurrence in China, India, Nepal, and Brazil.^1^ Traditionally used in Asian medicine for treating snake bites, liver disorders, and gastrointestinal conditions and promoting hair growth, it also finds use in Brazil for managing respiratory issues such as cough and asthma.^2,3^ Its pharmacological potential includes antioxidant, antimicrobial, hepatoprotective, anticancer, and neuroprotective effects, attributed to compounds such as phenolics, flavonoids, alkaloids, and steroids.^1,4^

**Figure 1:**
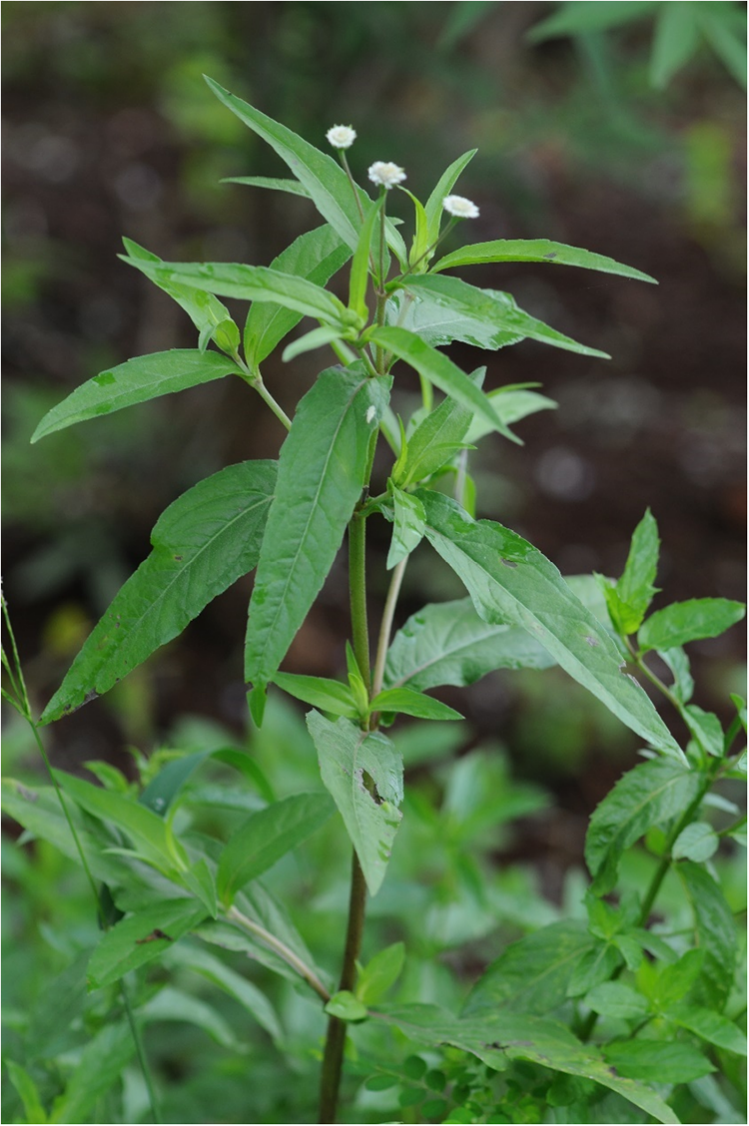
*Eclipta prostrata* plant.

Secondary metabolism in plants is modulated by both biotic and abiotic factors, including light, temperature, water availability, soil composition, and interspecific interactions.^5,6^ UV–B radiation, in particular, induces the synthesis of photoprotective secondary metabolites, such as flavonoids and tannins, which act as defense compounds under stress conditions.^7^ Similarly, nutrient availability in soils plays a key role; phenolic compound levels tend to increase in nutrient-poor soils, whereas nitrogen-rich environments often favor nitrogenous metabolites like alkaloids and glucosinolates.^8,9^

Biological interactions also modulate the secondary metabolism. Different plants cultivated in the same area can influence the quality of organic matter in the soil,^10^ and nitrogen fixation through plant–bacteria interactions or intercropping with legumes, such as *Arachis spp.*, enhances soil fertility and plant development.^11–13^ *Arachis repens* is used as a forage crop and for ornamental and ground cover,^14^ and it can enhance nitrogen fixation.^13^ However, studies exploring its use as green manure and its influence on metabolite production remain scarce. Additionally, plant–plant associations may trigger allelopathic effects via the release of phenolics, terpenes, and alkaloids, influencing the biosynthesis of secondary metabolites in cocultivated species.^12,15^

Given the ethnopharmacological relevance of *E. prostrata* and the influence of environmental and biological factors on its secondary metabolism, this study aimed to investigate how light exposure, soil type, and plant association affect biomass production and the accumulation of secondary metabolites in *E. prostrata*. Plants were cultivated under controlled conditions with different soil types, shade net presence, and intercropping with *A. repens* or intraspecific cultivation. The results provide insights into how these factors modulate the phytochemical profile of the plant, contributing to a better understanding of cultivation strategies for optimizing bioactive compound production.

## Materials and Methods

### Plant Material

Specimens of *E. prostrata* were obtained from “Farmácia da Natureza” from “Casa Esprita Terra de Ismael” located in Jardinópolis, SP, Brazil, coordinated by Prof. Dra. Ana Maria Soares Pereira (Universidade de Ribeirão Preto, Ribeirão Preto, SP, Brasil). Access to genetic resources was conducted in accordance with Brazilian legislation (13.123/2015), under SISGEN registration number ABBB366.

The identification of *E. prostrata* was carried out by Dr. Aristônio Magalhães Teles from the Department of Botany at the Institute of Biological Sciences, Federal University of Goiás (Goiânia, GO, Brazil) and that of *A. repens* was carried out by Milton Groppo Júnior from the Department of Biology, Faculty of Philosophy, Sciences and Letters of Ribeirão Preto-USP. A voucher specimen has been deposited in the Medicinal Plant Herbarium of UNAERP under the registration numbers HPMU-848 and HPMU 362, respectively.

### Plant Cultivation and Extract Preparation

Initially, genetically identical clones were prepared from stem cuttings of a single mother plant and cultivated in Carolina soil. After clones’ growth, the experimental treatments were applied and are summarized in Table 1. The treatments involved variations in the soil type, light exposure, and plant association. The shade net reduced light exposure by 70%. Carolina soil consisted of a commercial substrate composed of peat, vermiculite, rice straw, limestone, and other components from third-party manufactured products. Cerrado soil consisted of soil from a native *Cerrado* area without any additives. The soil substrate was a mixture of Cerrado soil, coffee husk, rice husk, and manure. In the association experiment, the control represented the standard cultivation of *E. prostrata* in the soil substrate without a shade net, serving as the reference for the biotic association treatments. For the abiotic stress experiment, comparisons were made with plants grown under natural conditions, where *E. prostrata* typically grows in Cerrado soil under light exposure. Therefore, plants cultivated in Cerrado soil without a shade net were considered the control for the abiotic treatments.

**Table 1:** Treatment Descriptions and Abbreviations for Biotic and Abiotic Stress Experiments on *E. prostrata* ^a^

treatment	stress	soil	shade net	association type
control	none	soil substrate	no	none
ExA	biotic	soil substrate	no	*A. repens*
ExE	biotic	soil substrate	no	*E. prostrata*
CL	abiotic	Carolina soil	no	none
CS	abiotic	Carolina soil	yes	none
SL	abiotic	Cerrado soil	no	none
SS	abiotic	Cerrado soil	yes	none
SBL	abiotic	soil substrate	no	none
SBS	abiotic	soil substrate	yes	none

a ExA: *E. prostrata* cocultivated with *A. repens*; ExE: *E. prostrata* cocultivated with another *E. prostrata*; CL: *E. prostrata* cultivated in Carolina soil without a shade net; CS: *E. prostrata* cultiavated in Carolina soil with a shade net; SL: *E. prostrata* cultivated in Cerrado soil without a shade net; SS: *E. prostrata* cultivated in Cerrado soil using a shade net; SBL: *E. prostrata* cultivated in the soil substrate without a shade net; SBS: *E. prostrata* cultivated in the soil substrate with a shade net.

The plants were cultivated in these conditions in 1 L pots under acclimated greenhouse conditions, with temperatures ranging from 22 to 28 °C for 3 months and then aerial parts were harvested and evaluated for height (cm), number of shoots, and fresh biomass (g). Samples were dried in a forced-air oven at 40 °C for 48 h, and the dry biomass (g) was measured. The dried plant material was ground using an IKA A11 analytical mill (USA) and stored at room temperature until further analysis. Abiotic treatments had 7 replicates each, and biotic treatments had 8 replicates each.

### LC-MS Analysis

The samples were prepared according to a methodology previously described.^16^ 50 mg of dried material was solubilized in 1 mL of methanol/water (7:3) in Eppendorf tubes. The tubes were vortexed for 10 s, sonicated for 20 min, and centrifuged for 10 min at 13,000 rpm. After centrifugation, the samples were treated with 200 μL of hexane twice for cleanup, filtered through 0.2 μm PTFE filters, and transferred to glass vials. During sample preparation, the internal standard (I.S.) betamethasone was added at a concentration of 0.5 mg/mL for the subsequent calculation of the relative area (signal area/internal standard area).

The method was developed by using a high-performance liquid chromatography (HPLC) system (Shimadzu LC-20A) coupled with a UV-DAD detector (CBM20A, Shimadzu). The samples (5 μL) were injected into an RP C18 Kinetex core–shell column (100 mm × 2.1 mm × 2.6 μm) at 35 °C and eluted with water and methanol, both supplemented with 0.1% formic acid (FA). The flow rate was 0.3 mL/min, using the following gradients: 5–30% B (0–5) min, 30–50% B (5–25 min), 50–95% B (25–28 min), 95% B (28–30 min), 95–5% B (30–31 min), and 5% B (31–35 min). Chromatograms were recorded at a wavelength of 280 nm.

To characterize the chemical profile, a representative sample was selected and diluted 10× in distillated water to analyze with the same HPLC-DAD method coupled with an electrospray ionization source and a qTOF mass spectrometer (Shimadzu). Chromatograms were obtained in both positive and negative modes with the following parameters: MS 100–1500 *m*/*z*; MS/MS 80–1000 *m*/*z*; spray voltage: +3.5 kV (positive mode) and −3.0 kV (negative mode); interface temperature: 300 °C; desolvation line temperature: 250 °C; interface temperature: 300 °C; heat block temperature: 400 °C; nebulizing gas flow: 3 L/min; heating gas flow: 10 L/min; drying gas flow: 10 L/min; nitrogen used as the drying, nebulizing, and fragmentation gas. The mass spectra were visualized using LabSolutions software (Shimadzu). Blanks were injected before each mode.

### Soil Content Evaluation

Soil analysis was performed by the company Ribersolo (Ribeirão Preto, SP, Brazil), accredited by the National Institute of Metrology, Quality, and Technology (INMETRO). Analyses of macronutrients, micronutrients, and sulfur were conducted upon request according to usual methodologies.^17,18^

### Data Analysis

The chromatographic signals were integrated, and the relative areas of each signal to the I.S. were calculated (signal area/internal standard area). These relative areas were used to compare variations in each compound content among the treatments. The data obtained were subjected to analysis of variance (ANOVA) using the System for Statistical Analysis of Balanced Data (SISVAR, version 5.1).^19^ Mean comparisons were performed using the Scott–Knott test at a 5% significance level (*p* < 0.05).

Compounds corresponding to the major chromatographic signals integrated were annotated according to the accurate molecular masses, UV spectra, and MS fragmentation pattern obtained.

The relationship between soil composition and metabolite production was evaluated using a Pearson correlation analysis of each component from soil analysis and metabolite relative areas. A correlation-based principal component analysis (PCA) was performed to assess associations between specific soil variables and metabolite profiles. These multivariate analyses were conducted using RStudio software (version 4.5.0).

## Results

### Growth and Biomass

Soil type and use of a shade net did not significantly affect plant height. However, a higher number of shoots and greater fresh and dry biomass were observed in substrate soil light (SBL) treatment, followed by Carolina soil light (CL) treatment (Table 2), indicating that enriched soils promote biomass accumulation. Regarding biotic treatments, intraspecific association reduced growth and biomass accumulation, whereas the association with *A. repens* resulted in a development similar to that of the control group (Table 3).

**Table 2:** Growth, Biomass Formation, and Number of Shoots of *E. prostrata* Cultivated under the Influence of Abiotic Factors^a^

treatment	height (cm)	shoots	fresh matter (g)	dry matter (g)
CL	48.29 ± 5.52 a	6.43 ± 2.64 a	13.80 ± 8.06 b	3.32 ± 1.93 b
CS	57.43 ± 7.41 a	3.00 ± 0.82 b	10.73 ± 2.84 b	1.15 ± 0.36 d
SL	58.00 ± 10.39 a	2.57 ± 0.79 b	8.28 ± 1.64 b	2.32 ± 1.48 c
SS	43.29 ± 10.34 a	1.71 ± 0.95 b	7.53 ± 4.10 b	0.79 ± 0.45 d
SBL	58.29 ± 5.22 a	5.86 ± 0.69 a	24.75 ± 1.80 a	4.90 ± 0.44 a
SBS	51.57 ± 12.27 a	2.14 ± 1.68 b	11.73 ± 6.63 b	1.16 ± 0.66 d

a Means followed by the same letter do not differ significantly at the 5% probability level according to the Scott–Knott test; values are expressed as mean ± standard deviation (SD).

**Table 3:** Growth, Biomass Formation, and Number of Shoots of *E. prostrata* Cultivated under the Influence of Biotic Factors^a^

treatment	height (cm)	shoots	fresh matter (g)	dry matter (g)
control	59.86 ± 3.80 a	4.63 ± 0.92 a	20.03 ± 3.01 a	3.72 ± 0.72 a
ExA	55.75 ± 5.90 a	4.63 ± 0.74 a	17.06 ± 2.55 b	3.50 ± 0.58 b
ExE	52.06 ± 4.90 b	3.69 ± 0.95 b	10.73 ± 2.74 c	2.28 ± 0.81 c

a Means followed by the same letter do not differ significantly at the 5% probability level according to the Scott–Knott test; values are expressed as mean ± standard deviation (SD).

### LC-MS Analysis

The chromatographic profile remained consistent across all of the treatments. However, variations in signal areas were observed, suggesting potential quantitative differences in compound levels among the treatments. To estimate these variations, comparisons were made based on the relative area of each signal (signal area/internal standard area). The corresponding signals are indicated in the chromatogram (Figure 2) and the putative annotation of the compounds is presented in Table 4.

**Figure 2:**
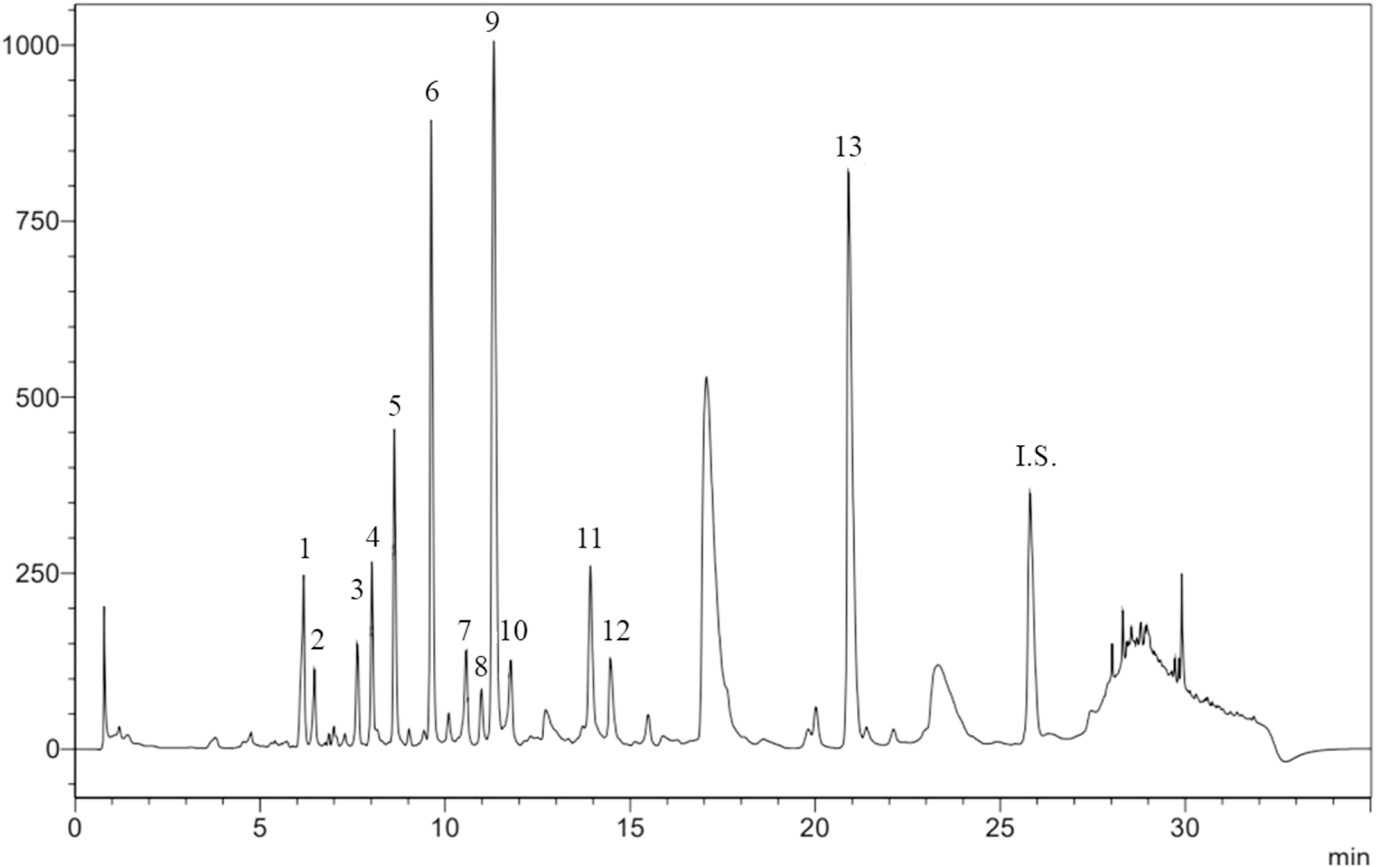
Representative chromatogram. I.S. indicates the internal standard.

**Table 4:** Chemical Constituents Annotated from *E. prostrata* by HPLC-DAD-MS^a^

peak	annotated compounds	RT (min)	[M + H]^+^	^+^MS^2^	[M – H]^−^	^–^MS^2^	UV_max_	molecular formula	error (ppm)
**1**	5-*O*-caffeoylquinic acid	6,17	355,10158	163,03884	353,08626	191,05458	290/325	C_16_H_18_O_9_	–2,20
**2**	3-*O*-caffeoylquinic acid	6,47	355,10121	163,03721	353,08616	191,05456; 179,03316; 173,04433	290/324	C_16_H_18_O_10_	–3,27
**3**	dihydroquercetin	7,63	305,06499	287,02920; 259,03800; 137,00200	UD	-	291/330sh	C_15_H_12_O_7_	–1,93
**4**	orobol-*O*-hexoside	8,03	449,10690	287,05408; 269,04404; 241,04873; 153,01742	447,09145	285,03889; 284,03244; 255,08758; 227,03398	260/290sh/325sh	C_21_H_20_O_11_	–2,09
**5**	myricetin-*O*-hexoside	8,62	481,09696	319,04395; 301,03520; 273,03937; 245,04273; 227,03468; 169,01214; 153,01784	479,08083	317,02812; 316,02074; 287,01841; 271,02481; 166,99649; 165,98980; 139,00210	259/275sh/356	C_21_H_20_O_13_	–1,47
**6**	quercetin-3-*O*-hexoside	9,64	465,10185	303,04916; 285,03858; 257,04414; 229,04870	463,08644	301,03358; 300,02499; 151,00288	281/344	C_21_H_20_O_12_	–1,93
**7**	isoluteolin-*O*-methyl-*O*-hexoside	10,59	463,12264	301,06991; 286,04587; 269,04354; 241,04844; 153,01733	461,10723	299,05306	260/290sh	C_22_H_22_O_11_	–1,84
**8**	3,4-di-*O*-caffeoylquinic acid	10,98	517,13170	163,03863	515,11728	353,08629; 335,07472; 191,05444; 179,03367; 173,04418; 135,04393	290/325	C_25_H24O12	0,04
**9**	3,5-di-*O*-caffeoylquinic acid	11,24	517,13273	163,03816	515,11777	353,08604; 191,05454; 179,03354; 135,04387	290/325	C_25_H_24_O_12_	–2,55
**10**	isoluteolin-*O*-methyl-*O*-hexoside	11,79	463,12271	301,06967; 286,04598; 269,04376; 241,04825; 153,01719	461,07060	298,01035	260/290sh	C_21_H_18_O_12_	–1,68
**11**	4,5-di-*O*-caffeoylquinic acid	13,90	517,13324	163,03815	515,11764	353,08587; 191,05460; 179,03360; 173,04404; 135,04399	290/328	C_25_H_24_O_12_	–1,57
**12**	demethylwedelolactone	14,36	301,03330	283,02468	299,01821	299,01812; 271,02322; 243,02899; 227,03362; 126,90356	248/351	C_15_H_8_O_7_	–3,26
**13**	wedelelolactone	20,95	315,04913	297,03824; 287,05408; 269,04311; 259,05848; 231,06513; 161,02259	313,03387	298,01017	248/351	C_16_H_10_O_7_	–2,54
**I.S.**	betamethasone (I.S.)	25,68	393,08626					C_22_H_29_FO_5_	

a RT: Retention time; [M + H]^+^: protonated molecule; ^+^MS^2^: product ion generated from the protonated molecule; [M – H]^−^: deprotonated molecule; ^–^MS^2^: product ion generated from the deprotonated molecule; UV: ultraviolet absorbance; UD: undetected.

Peaks **1, 2, 8, 9**, and **11** exhibited absorptions on the UV–vis spectra of UV_max_ ≈290 and 325 nm. These data, together with deprotonated ions obtained from mass spectra (peaks **1** and **2**: [M-H]^−^ = 353 and peaks **8, 9**, and **11**: [M – H]^−^ = 515) are characteristics of chlorogenic acids.^20^ Based on the product ion spectra, the peaks were identified as 5-*O*-caffeoylquinic acid (**1**), 3-*O*-caffeoylquinic acid (**2**), 3,4-di-*O*-caffeoylquinic acid (**8**), 3,5-di-*O*-caffeoylquinic acid (**9**), and 4,5-di-*O*-caffeoylquinic acid (**11**), according to the key identification criteria for chlorogenic acids previously proposed.^21–23^ These compounds were isolated and identified in *E. prostrata* before.^24–26^

Peaks **3–7** and **10** exhibited UV spectra and mass spectra, characteristic of different subclasses of flavonoid compounds. Except for peak **3**, all of the flavonoids presented a neutral loss of 162 Da, indicating a loss of a hexose group, compatible with flavonoids-*O*-hexosides.^27,28^

Peak **3** exhibited a UV_max_ = 290 and 330sh, characteristic of flavanones.^29^ The production ion spectra presented a loss of H_2_O [M+H-18]^+^ and CO [M+H-18–28]^+^ groups. It also presented a product ion of *m*/*z* 137, relative to fragment ^0,2^B^+^, a retro-Diels–Alder (RDA) product ion. Considering the spectra, the compound corresponding to peak **3** was tentatively assigned as dihydroquercetin (also known as taxifolin),^27,28,30^ which is endorsed by previous findings in the literature.^31^

Peaks **4, 7**, and **10** exhibited UV–vis spectra for the isoflavone subclass (UV_max_ = 260, 290, and 330sh).^29^ Peak **4** presented as the base peak of the aglycone *m*/*z* 287 [M+H-162]^+^ and a diagnostic product ion of *m*/*z* 153, relative to the fragment ^1,3^A^+^, a RDA product ion, that indicates two hydroxyl groups on the A ring.^27,28^ The product ion spectra also showed fragments such as *m*/*z* 269 [M+H-162–18]^+^ resulting from a loss of H_2_O from the aglycone *m*/*z* 241 [M+H-162–18–28]^+^ resulting from a sequence loss of a CO group. The fragmentation pattern and the UV spectra suggest an isoluteolin (also known as orobol) aglycone;^32,33^ then the compound corresponding to peak 4 was tentatively assigned as orobol-*O*-hexoside, which is endorsed by literature, being one of the main compounds found in *E. prostrata*.^1,34–36^

Peaks **7** and **10** presented the same ion product spectra, with the base peak being the aglycone at *m*/*z* 301 [M+H-162]^+^. The diagnostic ion product of *m*/*z* 153 is also present, indicating the A ring with two hydroxyl groups.^27,28^ The fragment ions of *m*/*z* 286 [M+H-15]^+^ results from a loss of a methyl group from the aglycone;*m*/*z* 269 [M+H-32]^+^ results from a loss of a CH_3_OH group; *m*/*z* 241 [M+H-32–28]^+^ results from a loss of a subsequent CO group. Considering the fragmentation pattern and the UV spectra, both compounds were tentatively identified as isoluteolin-*O*-methyl-*O*-hexoside.^29,33^ This is endorsed with previous findings in literature since these compounds were previously isolated and identified in *E. prostrata*.^35–39^

Peak **5** exhibited a UV spectra characteristic of flavonols (UV_max_ = 259 and 275sh and 354 nm).^29^ The mass spectra showed a neutral loss of 162 Da, indicating a loss of a hexose group, and the product ion ^1,3^A^+^ *m*/*z* 153 is also presented, indicating an *O*-hexose with two hydroxyl groups on ring A.^27,28^ The base peak is the aglycone *m*/*z* 319 and product ions resulting from a loss of CO [M+H-162–28]^+^ and H_2_O [M+H-162–18]^+^ are also present. With the mass spectra obtained in both negative and positive modes and the UV spectra, this compound was tentatively assigned as myricetin-*O*-hexoside, which is endorsed by the previous findings of this compound in *E. prostrata* extracts.^24,40,41^

Peak **6** presented a UV spectra typical of 3-OH-substituted flavonols (UV_max_ = 281 and 344 nm).^29^ The base peak was aglycone *m*/*z* 303 after a neutral loss of 162 Da of the hexoside group. Losses of CO [M+H-28]^+^ and H_2_O [M+H-18]^+^ are observed in the positive mode spectra and in the negative mode spectra, the diagnostic ion of ^1,3^A^–^ *m*/*z* 151 is the most intense peak after the aglycone, indicative of the A ring disubstituted with two hydroxyl groups. According to the UV and mass spectra, this compound was tentatively assigned as Quercertin-3-*O*-hexoside,^42^ which is corroborated with previous findings of this compound in *E. prostrata* extracts.^1,35^

Peaks **12** and **13** both presented UV_max_ values of 248 and 351 nm, with *m*/*z* 299 and 313 [M-H]^−^, respectively. Peak **12** mass spectra presented a base peak of *m*/*z* 283 [M-H_2_O–H]^−^ on positive mode, while on negative mode, the base peak was 271 [M-H_2_O–H]^−^. Peak **13** presented the base peak of *m*/*z* 298 [M-H–CH_3_]^−^ on negative mode, representing a loss of a methyl group. On positive mode, the base peak was *m*/*z* 287 [M-H–CO]^−^ and a secondary product ion *m*/*z* 297 [M–H-H_2_O]^−^. According to UV and mass spectra, these compounds were tentatively identified as demethylwedelolactone (**12**) and wedelolactone (**13**), respectively.^3,24,43,44^ These findings corroborate with literature data since wedelolactone and its derivative demethylwedelolactone are compounds commonly found in *E. prostrata*, especially wedelolactone, which is a marker compound responsible for biological activities.^3,24,34,35,44–52^

Data regarding the accumulation of each compound are presented in Figure 3.

**Figure 3:**
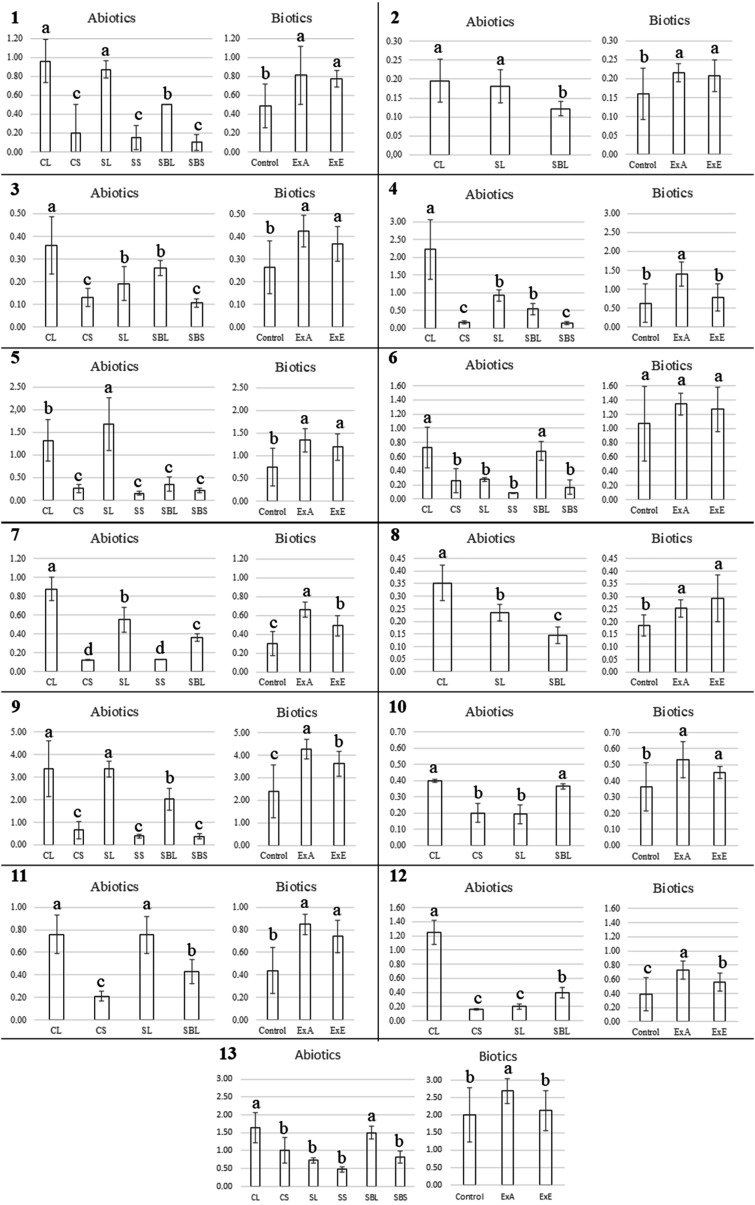
Comparison of the relative signal areas among the treatments. Each signal was designated as a compound by its respective number. Abiotic factors: comparison between soil types and the presence or absence of a shading mesh, as follows. CL: Carolina soil under light; CS: Carolina soil with shading; SL: Cerrado soil under light; SS: Cerrado soil with shading; SBL: substrate soil under light; SBS: substrate soil with shading. Biotic factors: comparison among the control group, *E. prostrata* associated with *A. repens* (ExA), and *E. prostrata* associated with *E. prostrata* (ExE). Results followed by the same letter do not differ significantly at the 5% probability level, according to the Scott–Knott test.

In general, treatments with shade nets (CS, SS, and SBS) showed lower metabolite accumulation compared to the groups with normal light exposure (CL, SL, and SBL), suggesting that the use of shade nets tends to reduce metabolite production.

Carolina soil and Cerrado soil increased metabolite accumulation of chlorogenic acids (**1, 2**, **8, 9**, and **11**) and the glycosylated flavonoids myricetin-*O*-hexoside (**5**) and isoluteolin-*O*-methyl-*O*-hexoside (**7**) compared to the substrate soil. However, substrate and Carolina soils promoted the accumulation of coumestans (**12** and **13**) and the glycosylated flavonoids quercetin-3-*O*-hexoside (**6**) and isoluteolin-*O*-methyl-*O*-hexoside (**10**). In general, Carolina soil stood out due to its consistent accumulation of all compounds, which suggests that Carolina soil provides the best conditions for the highest production of *E. prostrata* metabolites.

Plant association significantly enhanced metabolite accumulation. Both associations increased metabolite production compared to the control group, as evidenced for chlorogenic acids (**1, 2, 8, 9**, and **11**), flavonoids (**3, 4, 5, 7**, and **10**), and coumestans (**12** and **13**). When comparing the two types of association, the interspecific association outperformed the intraspecific type in accumulating orobol-*O*-hexoside (**4**), isoluteolin-*O*-methyl-*O*-hexoside (**7**), 3,5-di-*O*-caffeoylquinic acid (**9**), and the coumestans demethylwedelolactone and wedelolactone (**12** and **13**).

In summary, the treatment using Carolina soil with light exposure and the association with *A. repens* were the most effective in achieving higher secondary metabolite accumulation in *E. prostrata*.

### Soil Analysis

The physicochemical characterization of the soils (Table 5) presented different nutrient profiles. Cerrado soil presented the lowest macronutrient levels (OM, P, K, Ca, Mg, and S) and an intermediate profile of micronutrients (B, Cu, Fe, and Mn), and the S content and pH were similar to those of Carolina soil. The substrate soil exhibited the lowest pH and S content and higher levels of micronutrients (B, Cu, Fe, Mn, and Zn) and macronutrients (K and Ca). Carolina soil presented a higher amount of organic matter and the macronutrients P, Mg, and S. Except for B and Zn, it also presented the lowest levels of micronutrients Cu, Fe, and Mn.

**Table 5:** Physicochemical Characterization of Experimental Soils^a^

soil	pH	OM	P	K	Ca	Mg	H+Al	Al	S	SB	CTC	V%	m%	B	Cu	Fe	Mn	Zn
Cerrado	6,0	22	15	4,7	31	6	18	<1	53	41,7	60	70	1	0,17	2,2	10	13,5	0,6
substrate	5,3	50	85	47,4	51	25	21	<1	28	123,4	144	86	0	0,61	3,4	16	41,1	3,6
Carolina	6,1	74	137	10,8	15	53	9	<1	56	78,8	88	90	0	0,45	1,2	5	8,8	2,2

a Values represent analytical results for key parameters: pH, organic matter: OM (g/dm^3^), phosphorus: P (mg/dm^3^), potassium: K (mmolc/dm^3^), calcium: Ca (mmolc/dm^3^), magnesium: Mg (mmolc/dm^3^), potential acidity: H+Al (mmolc/dm^3^), aluminum: Al (mmolc/dm^3^), sulfur: S (mg/dm^3^), sum of bases: SB (mmolc/dm^3^), cation exchange capacity: CEC (mmolc/dm^3^), base saturation: V% (%), aluminum saturation: m% (%), boron: B (mg/dm^3^), copper: Cu (mg/dm^3^), iron: Fe (mg/dm^3^), manganese: Mn (mg/dm^3^), zinc: Zn (mg/dm^3^).

As shown in the Pearson correlation matrix (Figure 4) and principal component analysis (PCA), which revealed two principal components explaining 86.5% of total variance (PC1 = 49.5%, PC2 = 37%) (Figure 5), the accumulation of chlorogenic acids (**1, 2, 8, 9**, and **11**) is positively correlated with less acid pH and higher sulfur (S) content, while potassium (K), calcium (Ca), copper (Cu), iron (Fe), and manganese (Mn) presented negative correlations. Therefore, the production of these compounds is higher in Carolina soil and Cerrado soil. The coumestan (**12** and **13**) production was modulated by a significant positive correlation with organic matter, phosphorus (P) and magnesium (Mg), leading to a higher accumulation of these compounds in Carolina soil and the substrate soil. In contrast, flavonoids presented a more variable pattern.

**Figure 4:**
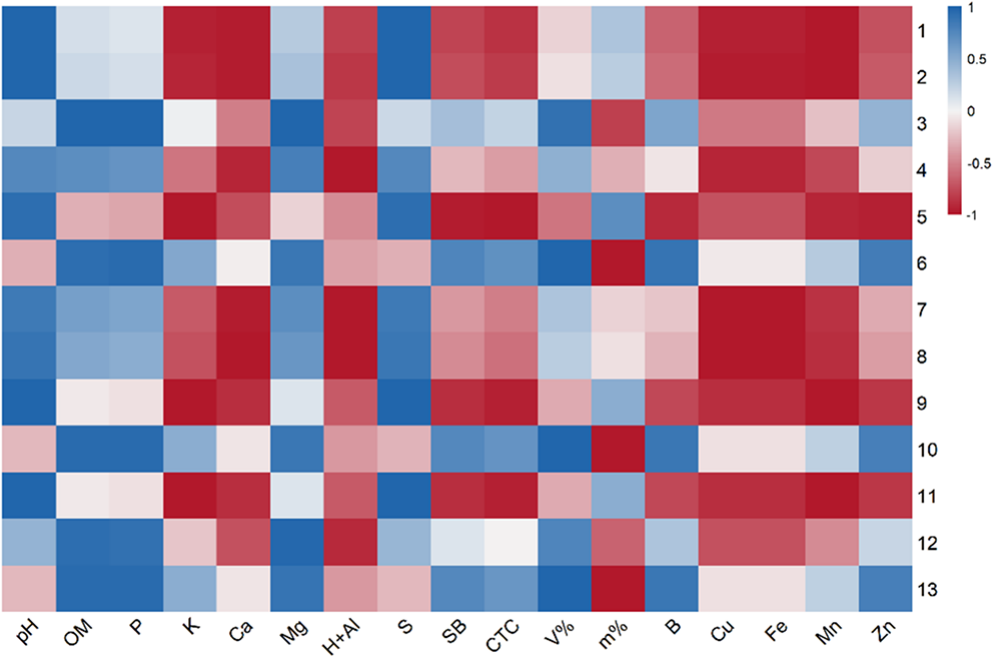
Pearson’s correlation heatmap between major secondary metabolites of *E. prostrata* and soil physicochemical parameters. Each row represents one identified compound, and each column represents one soil variable. Positive correlations are represented in blue tones and negative correlations in red tones, with the color intensity proportional to the strength of the correlation. Correlation (r) values close to 1 or −1 indicate strong relationships.

**Figure 5:**
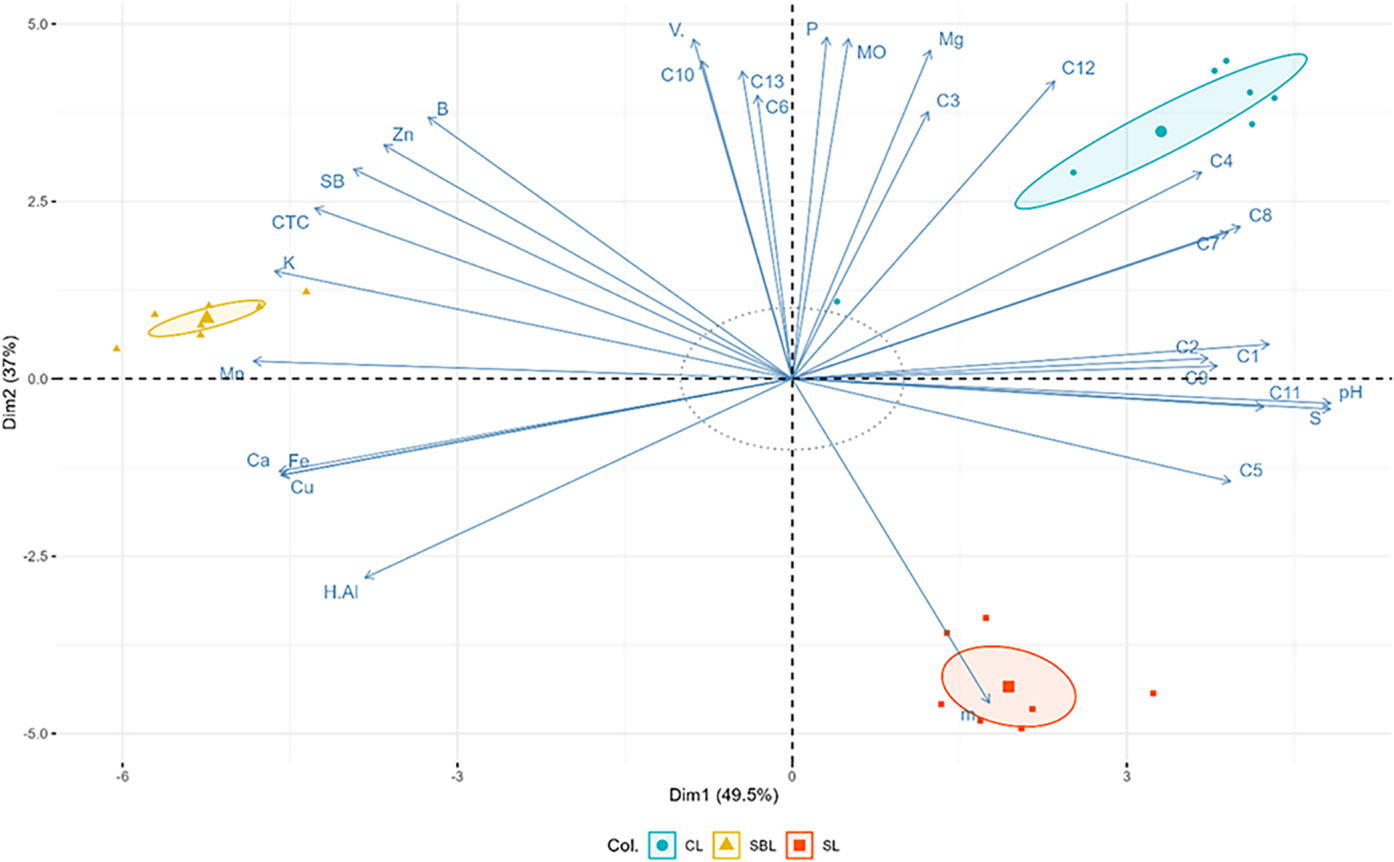
PCA biplot of soil components and compound relationship. C1–C13 represent each compound; soil parameters are abbreviated accordingly from previous nomination; CL = Carolina soil; SBL = substrate soil; SL = Cerrado soil; vector length indicates effect strength.

## Discussion

The ability of plants to adjust their metabolism in response to environmental variations is crucial for their survival and reproductive success, allowing metabolic adjustments that can vary according to the nature and intensity of abiotic and biotic stressors, as well as among different species and genotypes.^53^ As sessile organisms, plants rely on this metabolic flexibility to modify the synthesis of specialized metabolites that mediate defense, adaptation, and ecological interactions.^54^ This adaptability results from intrinsic biosynthetic plasticity and the regulation of gene expression, since stress conditions trigger specific genes that reprogram cellular metabolism, redirecting resources toward survival.^55^

An increase in plant biomass is often associated with an increase in the total amount of secondary metabolites.^8^ However, this was not observed in the present study since the substrate soil presented the highest biomass accumulation and the lowest production of most of the secondary metabolites. Plants cultivated in Carolina soil and Cerrado soil had similar biomass yields and higher metabolite production, indicating that the variations in metabolite levels were not directly related to biomass but rather to the different types of soils/substrates and light exposure. This was also observed in association treatments since association decreased biomass accumulation but increased secondary metabolite production compared to the control. Therefore, the variation is due to the influence of plant association on secondary metabolism rather than biomass accumulation.

The treatments with shading (CS, SS, and SBS) had a lower metabolite accumulation, which may be attributed to the influence of UV–B radiation on plant metabolism. UV–B radiation favors the production of phenolic compounds, which protect the plant from the damaging effects of solar radiation.^7,8^ Flavonoids, chlorogenic acids, and coumestans were the main compounds found in this study, and the increase in the production of these metabolites is regulated by enzymes of the phenylpropanoid biosynthetic pathway, whose gene expression is enhanced by UV–B exposure.^8,56^

Soil composition was another important factor related to secondary metabolite production in this study. While nutrient addition is commonly used to increase biomass, the effects of soil type on secondary metabolism are not entirely predictable and vary depending on environmental conditions. However, there is a tendency for nutrient-poor soils to show lower biomass accumulation and higher production of secondary metabolites, particularly phenolic compounds.^8^ This can be observed when comparing Cerrado soil with the substrate soil, which showed greater biomass but less accumulation of chlorogenic acids.

Although soil effects on secondary metabolism are unpredictable, in this study, certain soil components presented patterns related to the compounds annotated. The chlorogenic acids were strongly positively correlated with the pH and sulfur content. While the pH is directly related with nutrient availability,^57^ sulfur is an essential macronutrient with a high potential for inducing pathogen resistance in plants.^58–60^ Studies showed that sulfur can increase phenolic compound accumulation.^59–61^ Sulfur fertilizers can increase chlorogenic acid production,^62^ and field studies with *Brassica napus* demonstrated that adding sulfur to the soil in combination with organic fertilizers resulted in higher levels of chlorogenic acid.^63^

In contrast, coumestans (demethylwedelolactone and wedelolactone) were strongly positively correlated with organic matter, potassium, and magnesium, being more influenced by enriched soils like Carolina soil and the substrate soil. This is the second report associating these compounds with soil nutrition.^64^

The flavonoids presented more variable results in response to the soil composition. The flavonoids orobol-*O*-hexoside, myricetin-*O*-hexoside, and isoluteolin-O-methyl-O-hexoside (**7**) positively correlated with the pH and sulfur, similar to the chlorogenic acids, possibly due to phenylpropanoid pathway activation.^59–61^ Dihydroquercetin, quercetin-3-*O*-hexoside, and isoluteolin-*O*-methyl-*O*-hexoside (**10**) positively correlated with organic matter, potassium, and magnesium. These findings align with a previous study in which certain flavonoids, including dihydroquercetin, presented a positive correlation with organic matter.^65^ In the same study, manganese was negatively correlated with some phenolic compounds,^65^ as found in the present work. The variability in flavonoid accumulation suggests a more complex relationship between these compounds and soil composition.

Regarding the association, both interspecific and intraspecific cultivation increased metabolite production. *A. repens* enhances nitrogen fixation, providing a source of nutrients.^13^ Interspecific cultivation can increase the enzymatic activity of enzymes related to the phenyphonanoid pathway,^59^ which may lead to an increased synthesis of allelochemicals such as phenolic compounds and flavonoids.^12^ The intraspecific treatment can also involve allelopathy since *E. prostrata* is capable of using allelopathic mechanisms, releasing flavonoids and coumestans.^15^

## Conclusion

The findings of this study demonstrate that secondary metabolism in *E. prostrata* is responsive to both abiotic and biotic stimuli. Light exposure emerged as a major factor promoting the synthesis of the studied metabolites. Plant associations, particularly with *A. repens*, also enhanced the metabolite accumulation. Soil composition played an important role under the studied conditions, with the pH and sulfur content positively influencing chlorogenic acid production and organic matter favoring coumestan accumulation. Among the tested conditions, Carolina soil provided the most favorable environment for the studied secondary metabolite production. These insights emphasize the importance of integrating soil management, light exposure, and plant interactions to optimize the cultivation of *E. prostrata* for medicinal or agricultural applications.
